# NGAL Correlates with Femoral and Carotid Plaque Volume Assessed by Sonographic 3D Plaque Volumetry

**DOI:** 10.3390/jcm9092811

**Published:** 2020-08-31

**Authors:** Michael Schreinlechner, Maria Noflatscher, Daniela Lener, Axel Bauer, Rudolf Kirchmair, Peter Marschang, Markus Theurl

**Affiliations:** 1 Medical University of Innsbruck, University Hospital of Internal Medicine, Cardiology and Angiology, Anichstrasse 35, A-6020 Innsbruck, Austria; Michael.Schreinlechner@i-med.ac.at (M.S.); Maria.Noflatscher@i-med.ac.at (M.N.); Daniela.Lener@tirol-kliniken.at (D.L.); Axel.Bauer@i-med.ac.at (A.B.); Rudolf.Kirchmair@i-med.ac.at (R.K.); Peter.Marschang@i-med.ac.at (P.M.); 2 Central Hospital of Bolzano, Department of Internal Medicine, Via Lorenz Boehler 5, I-39100 Bolzano, Italy

**Keywords:** atherosclerosis, ultrasound, biomarker, preventive medicine

## Abstract

Background/Objectives: Inflammation represents a cornerstone in the development of atherosclerosis and early detection is essential to avoid cardiovascular events. Biomarkers like interleukin-1 beta, interleukin-6, or high sensitivity CRP (hs-CRP) have been investigated intensively in this field. Since they have several limitations, additional biomarkers are needed for cardiovascular risk stratification. The acute phase protein, neutrophil gelatinase-associated lipocalin (NGAL), modulates inflammation and is elevated in cardiovascular disease (CVD). Moreover, it contributes to plaque destabilization. Methods: In this prospective, single-center study, we included 323 asymptomatic patients with at least one cardiovascular risk factor or established CVD. NGAL levels were measured in plasma samples using a commercially available ELISA. Carotid, femoral, and total atherosclerotic plaque volumes (PV) were measured using a 3D ultrasound system (Philips iU22). Patients were separated into a low (*n* = 243) and high (*n* = 80) total PV group. Results: NGAL was significantly higher in patients with high total PV versus patients with low total PV. The NGAL amplitude for the prediction of high total PV was significantly higher when compared with hs-CRP. A high predictive value could also be observed for patients without established CVD. Conclusion: NGAL seems to be a promising biomarker for the identification of asymptomatic patients with atherosclerotic disease.

## 1. Introduction

Cardiovascular disorders represent the major cause of death in the industrialized world. Arterial hypertension, hypercholesterolemia, diabetes mellitus, and nicotine consumption have been identified as strong risk factors for the development of atherosclerotic lesions. Recent data suggest that atherosclerosis is driven by chronic vascular inflammation and that ongoing inflammation within plaques is associated with plaque vulnerability and disruption [1,2]. Elevated levels of high sensitive C-reactive protein (hs-CRP) have been associated with an increased risk of cardiovascular events and underline the hypothesis of atherosclerosis being an inflammatory disorder [3].

To identify patients at high risk for cardiovascular events, the European Society of Cardiology recommends the use of risk scores [4]. What these scores have in common is that classical risk factors are included in their algorithms. Biomarkers have not yet been included in these scores, although they may have the potential to specify individual cardiovascular risk. This approach may allow preventive actions to avoid future cardiovascular events.

Neutrophil gelatinase-associated lipocalin (NGAL) is a 25 kilo Dalton (kDA) acute phase protein belonging to the lipocalin superfamily and was initially described as a biomarker for acute and chronic renal failure [5]. Recent clinical trials reported elevated NGAL plasma levels also in coronary artery disease, myocardial infarction, and heart failure [6]. These findings are not only derived from animal experimental data but have also been confirmed in clinical studies [7,8]. For example, Soylu and co-workers found elevated NGAL levels in patients presenting with non-ST segment elevation myocardial infarction [9]. Moreover, NGAL levels correlated with established cardiovascular scores like the global registry of acute coronary events (GRACE) risk score and showed a positive association with the synergy between percutaneous coronary intervention with taxus and cardiac surgery (SYNTAX) score [9]. In addition, NGAL seems to be a predictor of all-cause mortality in ST segment elevation myocardial infarction (STEMI) patients and positively correlates with the severity of coronary heart disease [10,11]. Interestingly, in a Danish registry, the measurement of NGAL in addition to other biomarkers and cardiovascular risk factors did not add any predictive value in a high risk population with stable coronary heart disease [12].

Experimental data revealed that NGAL forms a complex with matrix metalloproteinase-9 (MMP-9), thereby prolonging the proteolytic activity of MMP-9 and contributing to plaque vulnerability. This could lead to plaque rupture and subsequently, to a cardiovascular event. In addition, vascular stability could be modified by NGAL. Animal experimental data show that mice are less prone to aneurysm formation after NGAL knockout [13].

Recently, it has been postulated that adipose tissue is a reservoir of inflammation in obese patients. In accordance with these data, a significantly increased expression of NGAL could be detected in the visceral fatty tissue of obese patients. Congruently, a correlation of serum NGAL level with insulin resistance could be shown [14]. These data are concordant with the observations of Eilenberg and co-workers, who found elevated plasma NGAL levels in diabetics [15]. Interestingly, NGAL levels could be influenced by the intake of metformin [15]. A decrease in NGAL plasma levels was also observed after statin intake, consistent with the hypothesis that NGAL modulates inflammation, as shown by the upregulation of interleukins and monocyte chemoattractant protein-1 in vascular cells and the anti-inflammatory properties of statins [16,17].

According to the abovementioned characteristics of NGAL, we hypothesized that this biomarker may be altered also in patients with peripheral atherosclerotic disease and therefore, may be a valuable biomarker to guide patient management in preventive medicine.

## 2. Methods

### 2.1. Study Design

Patients were recruited from the prospective observational single-center cohort study “Correlation of Atherosclerotic Plaque Volume and Intima Media Thickness With Soluble P-selectin trial” (Clinical trials.gov identifier: NCT01895725; approved by the ethics committee of the Medical University of Innsbruck on 31/May/2013, approval number UN5048). The baseline results of this trial have been reported [18].

Briefly, individuals aged 30 to 85 years with established CVD (coronary artery disease (CAD), cerebrovascular disease, peripheral arterial occlusive disease (PAD) or at least one traditional cardiovascular risk factor (CVRF; arterial hypertension, smoking, hyperlipidemia, diabetes, family history of cardiovascular disease) were included. A sonographic examination with automated measurement of the intima media thickness (IMT) with a linear L9-3 probe and quantification of the atherosclerotic plaque volume with a VL13-5 3D probe were performed using the Philips iU22 ultrasound system (Amsterdam, The Netherlands). A detailed description of the ultrasound examination is given below. Plaques were defined according to the Mannheim consensus [19]. In addition, all participants underwent ankle brachial index (ABI) and pulse wave velocity determination measured by an automated system (AngE Pro 4, SOT Medical Systems, Maria Rain, Austria).

Laboratory analysis included total cholesterol, HDL cholesterol, LDL cholesterol, triglycerides, hs-CRP, fasting glucose, hemoglobin A1c, creatinine, and estimated glomerular filtration rate (eGFR). For 342 patients, an additional EDTA blood sample was centrifuged at 1730× *g* and the plasma was stored at −80°. The level of NGAL was measured using a commercially available ELISA (BioPorto, Kit 036RUO Human NGAL).

**Figure 1:**
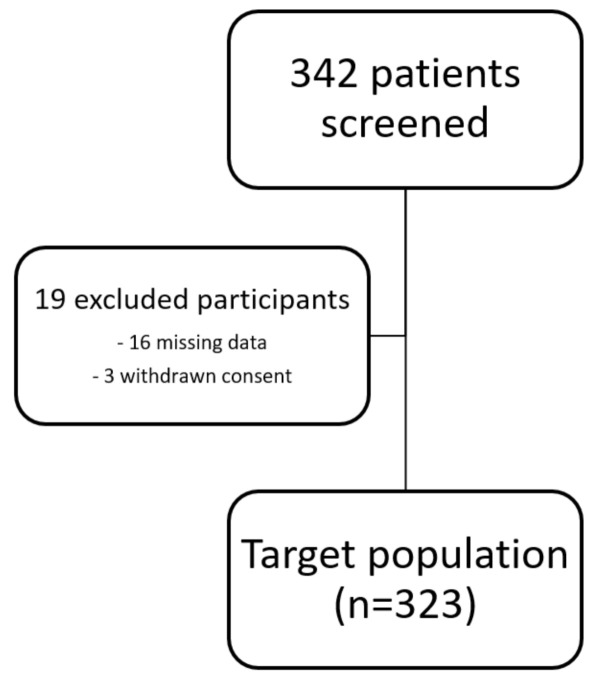
Flow chart of study participants.

The study protocol has been approved by the Ethics Committee of the Medical University of Innsbruck and complies with the Declaration of Helsinki. All patients gave written informed consent before inclusion into the study. Due to missing data and withdrawn consent of some patients, 323 participants were included in the final analysis (see Figure 1).

Family history of CVD was defined according to currently practiced recommendations as the occurrence of a premature cardiovascular event (myocardial infarction, stroke, or critical limb ischemia) in a first-degree relative (<55 years for male and <65 years for female relatives). The eGFR was calculated using the Modification of Diet in Renal Disease (MDRD) formula and chronic kidney disease (CKD) was defined as estimated glomerular filtration rate (eGFR) < 60 mL/min/1.73 m^2^. For diabetes, a fasting glucose level ≥ 126 mg/dL or the use of diabetes medication was considered relevant. Arterial hypertension was defined as systolic blood pressure ≥ 140 mmHg and/or diastolic blood pressure ≥ 90 mmHg and/or the use of antihypertensive therapy. Hyperlipidemia was defined as a low-density lipoprotein value ≥ 160 mg/dL and/or triglycerides ≥ 150 mg/dL and/or use of lipid lowering drugs.

### 2.2. Ultrasound Examination

Each participant underwent a routine sonographic examination measuring the flow velocity of the common carotid artery, the internal carotid artery, the external carotid artery, and the vertebrate artery, as well as the common femoral artery, the proximal superficial femoral artery, and the deep femoral artery. In addition, we performed measurements of IMT according to the recommendations of the Mannheim Consensus in the far wall of the distal carotid artery, as well as the proximal superficial femoral artery 1 cm distal to the flow divider along a 10 mm segment free of plaques [19]. For IMT measurements, we used a Philips iU22 system (Philips, Amsterdam, The Netherlands) equipped with an L9-3 linear probe using integrated, automated IMT calculation software. The measurements were performed in end diastole. The plaque volume was defined as a local structure protruding at least 0.5 mm into the arterial lumen, or 50% of the surrounding IMT, or having a thickness > 1.5 mm, as measured from the media–adventitia interface to the intima–lumen interface. The plaque volume measurements were performed using the Philips iU22 ultrasound system (Philips, Amsterdam, the Netherlands) equipped with a 3D VL13-5 probe and plaque quantification software to evaluate the volume of the plaque on both sides. The plaque volume was measured over a distance of 6 cm in the bifurcation and the adjacent parts of the internal arteries and carotid artery, as well as in the common femoral artery, femoral bifurcation, and adjacent parts of the proximal superficial femoral artery.

To assess method reliability, interobserver variability of 3 different observers was calculated and showed a very good agreement between the raters with an intraclass correlation coefficient of 0.95 (95% CI, 0.82–0.99).

### 2.3. Statistical Analysis

To test for normal distribution, the Kolmogorov–Smirnov test was used. The baseline data for continuous variables are shown as mean ± standard deviation (SD) for normally distributed parameters or as median and corresponding interquartile range (IQR) for parameters not following a normal distribution. Categorical variables are displayed as absolute numbers and percentages. Subjects were analyzed according to total PV and separated into two groups: <75th percentile (low total PV) and ≥75th percentile (high total PV). A two-tailed, independent samples t-test or the Mann–Whitney U test were used to assess differences between continuous variables, as appropriate. The χ2 test was used to evaluate variations in categorical variables. Total PV was calculated adding the sum of carotid and femoral PV. To assess the linear correlations for selected variables, Pearson or Spearman test were used as appropriate.

To calculate the predictive value of NGAL for high total PV, receiver operating characteristics (ROC) were performed to determine the area under the curve (AUC) and the optimal cut-off values with the highest sensitivity and specificity [20]. AUC values were compared using a nonparametric approach [21].

Univariate and multivariate linear regression were used to analyze the relationship between the dependent variables (total PV) and the predictor variables (biomarkers, traditional CVRF). Only variables with *p* < 0.05 in univariate analysis were considered for multivariate regression.

Interobserver variability comparing 10 vessels for plaque volume quantification was determined by an intraclass correlation coefficient (ICC).

For all statistical tests, a two-tailed *p*-value of <0.05 was considered as significant. Statistical analyses were conducted with SPSS Statistic Version 24.0 (IBM Corp, Armonk, NY, USA) and MedCalc Version 15 (MedCalc Software, Ostend, Belgium).

## 3. Results

### 3.1. Study Population

In total, 323 patients (45% females), with a mean age of 64.2 ± 9.0 years, were included in the analysis and underwent 3D plaque volumetry of carotid and femoral arteries.

Detailed demographic, clinical, and laboratory characteristics of the study population are summarized in Table 1.

About one-third of the study participants suffered from established CVD, but overall, the study group belonged to an intermediate risk population with a median of two cardiovascular risk factors.

Hyperlipidemia (87.6%) and arterial hypertension (65.0%) were the most common CVRFs. One quarter of the study population had a family history of CVD (25.4%) and around 20% were smokers, while only a minority presented with diabetes (13%). A total of 81% of diabetics were treated with antidiabetics, with most of them receiving oral medication (83% of treated patients). The mean HbA1c of the diabetic patients was 7.2%.

The study population was divided according to the plaque burden in low (<75th percentile) and high total PV (≥75th percentile). Patients with high total PV were significantly older (*p* < 0.001), less frequently females (*p* < 0.001), and suffered, not unexpectedly, more often from established CVD (*p* < 0.001).

Half of the study population was treated with antihypertensive (57.6%) and lipid-lowering drugs (55.1%), with moderate and high intensity statins being taken more frequently by patients in the high PV group.

### 3.2. Plaque Volume, Intima Media Thickness, Ankle Brachial Index, and Pulse Wave Velocity

Median values for total PV, carotid PV, and femoral PV are displayed in Table 2. As expected, patients with a higher total PV had also a significantly higher carotid (*p* < 0.001) and femoral (*p* < 0.001) PV. In addition, the median pulse wave velocity, as well as mean carotid IMT and ankle brachial index, are shown.

ABI is known as strong indicator of generalized atherosclerosis and CV risk. An ABI ≤ 0.90 is associated, on average, with two to three times higher risk of total death and CV events [22,23]. In addition, ABI measurements can identify the patient’s risk for lower limb events.

As shown in Table 2, patients in the low volume PV group had a low normal ABI. In contrast, the ABI of the high PV group was 0.89, just below the cut-off for the presence of PAD. Although the difference between the groups was small, it was statistically significant. The ABI itself showed only a poor correlation with NGAL in the overall collective (R = −0.19, *p* < 0.001). Interestingly, in the diabetic cohort, a better correlation was found (R = −0.39, *p* = 0.012).

Moreover, patients in the high PV group showed increased pulse wave velocity compared to the low PV group. Similar to the ABI, pulse wave velocity is a marker of atherosclerosis and a predictor for cardiovascular events. Both the association with low ABI and high pulse wave velocity confirm that the patients in the high PV group represent a collective at higher cardiovascular risk.

### 3.3. Associations between NGAL and PV

**Figure 2:**
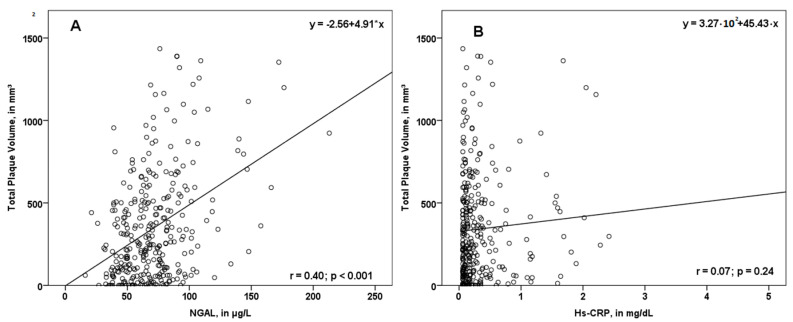
Scatterplot displaying the association of NGAL (**A**) and hs-CRP (**B**) with total plaque volume (PV). While NGAL was associated with total PV, hs-CRP did not correlate significantly with the total plaque volume. NGAL—neutrophil gelatinase-associated lipocalin; hs-CRP—high sensitivity C-reactive protein; r—correlation coefficient.

In our study, NGAL values were associated with total PV (r = 0.40; *p* < 0.001, Figure 2A), femoral PV (r = 0.27; *p* < 0.001), as well as carotid PV (r = 0.41; *p* < 0.001). Interestingly, the association between the biomarker hs-CRP and PV was not significant (Figure 2B).

### 3.4. Utility of NGAL for the Prediction of Higher PV

Patients with high PV showed significantly higher median NGAL values compared to patients with low PV (81.9 µg/L vs. 63.0 µg/L; *p* < 0.001).

**Figure 3:**
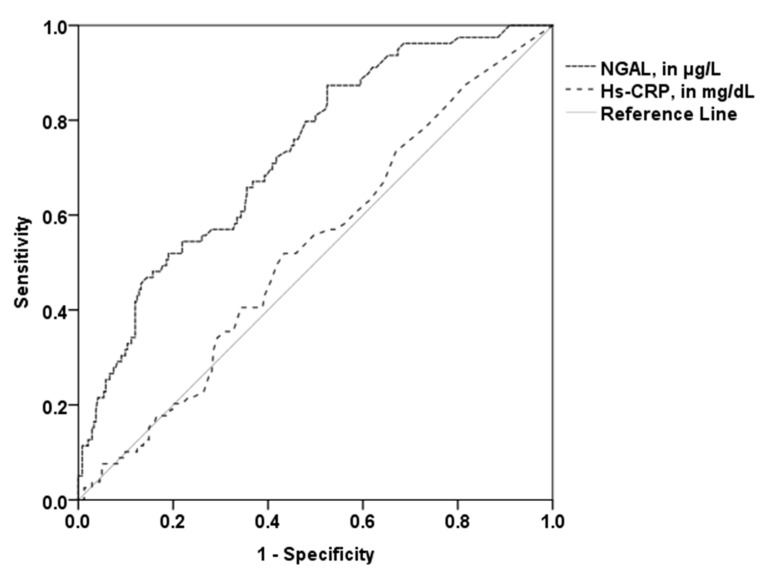
Receiver operating curves for the prediction of higher total PV. The AUC of NGAL (0.73) was significantly higher than the AUC of hs-CRP (0.53). NGAL—neutrophil gelatinase-associated lipocalin; hs-CRP—high sensitivity C-reactive protein.

The area under the curve (AUC) of NGAL (0.73, 95% CI 0.67–0.79; *p* < 0.001) with the optimal cut-off value of 81.18 µg/L revealed 52% sensitivity and 81% specificity in the prediction of higher PV. The AUC of hs-CRP (0.53; 95% CI 0.48–0.58; *p* = 0.45; optimal cut-off 0.19 mg/dL) was significantly lower compared to the AUC of NGAL (*p* = 0.001; Figure 3). The patient group with established CVD showed a similar AUC (0.75, 95% CI 0.66–0.84; *p* < 0.001). A high predictive value for NGAL could also be observed for patients without established CVD (AUC 0.69; 95% CI 0.58–0.78; *p* < 0.001). Conversely, the AUC of hs-CRP was not a significant predictor for high PV in these two subgroups.

**Figure 4:**
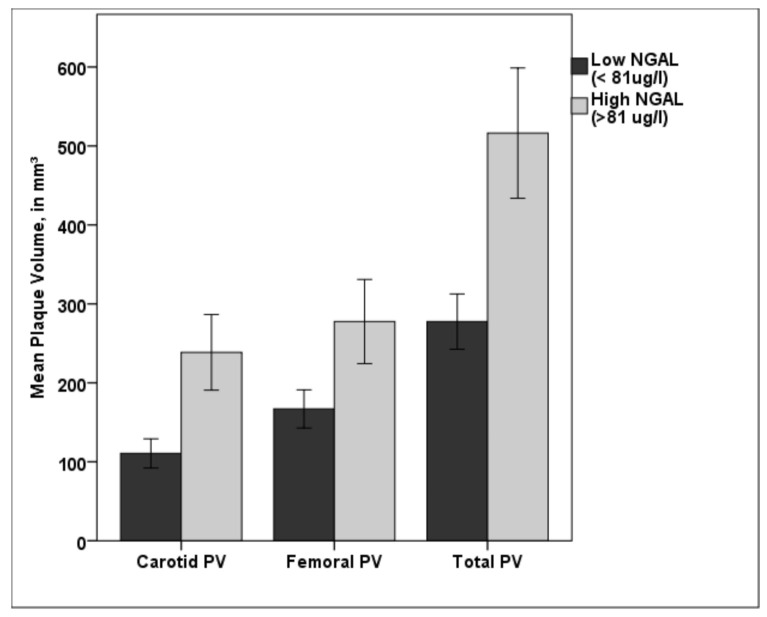
Bar diagram showing the difference in carotid, femoral, and total PV for low NGAL (<81.18 µg/L) and high NGAL (>81.18 µg/L). Patients in the high NGAL group showed a significantly higher plaque burden in both vascular beds compared with patients in the low NGAL group. PV—plaque volume; NGAL—neutrophil gelatinase-associated lipocalin; * *p* < 0.001. Error bars indicate 95% confidence intervals.

Patients with NGAL values over the cut off value of 81.18 µg/L showed significant higher carotid, femoral, and total PV (*p* < 0.001, respectively) than patients with lower NGAL values (see Figure 4).

### 3.5. Multivariate Analysis

Multivariate regression was calculated to predict total PV based on biomarkers and traditional CVRFs (see Table 3).

*P*-values < 0.05 in univariate analysis for total PV were observed for: age, established CVD, eGFR, male gender, hypertension, smoking, and NGAL. In contrast, no significant association could be found for antihypertensive, lipid lowering, or antiplatelet therapy.

In multivariate analysis, all included parameters showed a significant contribution. However, comparing regression coefficients, age (*b* = 0.40; *p* < 0.001), NGAL *(b* = 0.28; *p* < 0.001), and established CVD (*b* = 0.24; *p* < 0.001) were the strongest predictors for total PV.

## 4. Discussion

In this prospective, single-center study, we investigated the role of the acute phase protein NGAL on atherosclerosis in outpatients with at least one established CVRF or a manifestation of CVD.

Recent data strengthen the hypothesis that thrombus formation and the development of atherosclerosis is fueled by immune cells through the activation of coagulation and release of chemokines [24]. The clinical proof of concept of this hypothesis was recently published by Ridker et al., showing positive effects of the interleukin 1-ß-antibody canakinumab in patients with previous myocardial infarction in the CANTOS (Canakinumab Anti-inflammatory Thrombosis Outcome) study [3].

In a small study, Eilenberg and colleagues were able to detect NGAL in carotid plaques of patients undergoing carotid endarterectomy [17]. In their study, NGAL expression was more pronounced in tissue samples of patients with symptomatic carotid atherosclerosis, supporting the hypothesis that NGAL may be associated with active atherosclerosis [17]. Moreover, in vitro studies of the same group demonstrated a significant increase in proinflammatory cytokines like IL-6, IL-8, and MCP-1 from vascular cells treated with recombinant NGAL, indicating that NGAL might also be involved in the process of immunothrombosis [17]. In a follow-up work with 83 patients, the same authors were able to demonstrate that NGAL levels were higher in patients with vulnerable plaque morphology [25]. The results of our study support these findings and may contribute to a better understanding of NGAL in peripheral atherosclerosis. The novelty of our trial is that we provide data on an association of NGAL with 3D plaque volume assessment in the carotid, and for the first time, also in the femoral arteries.

The role of hs-CRP has been demonstrated in previous publications [3,26]. Interestingly, in our study, hs-CRP showed a lower correlation with plaque burden than NGAL. This might be due to several reasons. The abovementioned CANTOS trial investigated the role of canakinumab in patients with elevated hs-CRP after acute myocardial infarction [3]. In our study, patients with an acute event were not included and most of the patients had a low or intermediate cardiovascular risk according to current guidelines. Moreover, about fifty percent of patients were on therapy with statins that are known to lower CRP levels and therefore, may explain the lower correlation [26].

Interestingly, in our study, patients in the high PV group had lower LDL cholesterol levels. We know that NGAL levels are influenced by statins [27]. Although statin intake was balanced between the groups, we found in a more detailed analysis that patients in the high PV group were prescribed significantly more moderate or high intensity statins (Figure S1a). This finding may explain the difference in LDL cholesterol levels between the two groups and the missing difference in NGAL levels between patients on statin therapy and patients without statin intake (Figure S1b).

Since Eilenberg et al. also reported an influence of metformin on NGAL levels, we also analyzed metformin intake in our study population. We did not find statistically significant different NGAL levels in diabetic patients with high or low PV in our study. Moreover, metformin intake was balanced between the low and high plaque volume group. In our study, NGAL levels were comparable between patients receiving metformin and those who were metformin-naïve (Figure S2). However, we must admit that the absolute number of diabetics in our study was very low and this could be one reason why the findings of Eilenberg and collaborators were not reproduced in our study [16].

Interestingly, the high predictive value of NGAL for plaque burden was also observed for patients without established CVD, indicating that this protein might be a promising marker for atherosclerosis in patients with lower cardiovascular risk. The multivariate analysis identified age and NGAL as the strongest predictors for total PV, again, demonstrating that NGAL represents a selective biomarker for this disease.

NGAL is also secreted from renal tubular cells and is a biomarker for acute kidney injury [28]. Therefore, one might hypothesize that the higher NGAL levels in the “high plaque burden” group can be traced back to a higher number of patients with renal failure. Nevertheless, the eGFR did not differ between the groups.

In some patients, we evaluated levels of IL-1beta and IL-6 (Figure S3). However, in our study population, there was no difference between the low and high plaque volume group. Interestingly, these interleukins were not even detectable in some samples. In our opinion, this again reflects the fact that this is a cohort with an overall lower plaque load. Perhaps NGAL appears to be a more reliable biomarker in this population. Of course, this is very speculative and would, therefore, have to be confirmed in a larger multicenter study.

In this publication, we present the baseline data of our study. A follow-up over five years with 3D ultrasound and NGAL measurement is ongoing. Therefore, we hope that we can provide data on the association of NGAL with plaque progression in the near future. To reliably document plaque progression, we decided to perform sonographic 3D plaque volumetry, which is a practicable and new approach for the exact determination of plaque volume in peripheral and carotid arteries [18,29]. Recent studies showed a strong association between peripheral plaque measurement via sonographic 3D ultrasound and calcium score assessment by computed tomography [30]. The major advantages of 3D ultrasound are the lack of radiation for the patient, the low costs for the health system, and the broad availability of ultrasound.

To our knowledge, this is the first study demonstrating a correlation of NGAL levels with carotid and femoral plaque volume measured by 3D ultrasound. Considering the current literature and our data, we think that NGAL appears to be an interesting biomarker for risk assessment in patients with established CVD as well as in patients with subclinical atherosclerosis. The combination of classical risk scores with biomarkers like NGAL might be an innovative approach in preventive medicine.

### Limitations

The design of our study includes several limitations. Although more than 300 patients were included, which is more than in several previous investigations, the sample size is still relatively small. Additionally, the study was conducted at one center and we do not provide a control group. To confirm ours and former findings, a multicenter study with a matched control group without manifestation of atherosclerosis would be desirable. Finally, our population belonged to a low to intermediate risk population. Therefore, at the moment the results are probably not transferable to a high risk group.

## Figures and Tables

**Table 1:** Demographic, clinical, and biochemical characteristics for the overall study population, the group with lower total PV, and the group with higher total PV.

	Study Population (*n* = 323)	Low Total PV (*n* = 243)	High Total PV (*n* = 80)	*p*-Value
Age, years	64.2 (±9.0)	62.7 (±8.9)	68.6 (±7.7)	**<0.001**
Female, n (%)	144 (44.6)	122 (50.2)	22 (27.5)	**<0.001**
CVRF, n	2.0 (1.0)	2.0 (1.0)	3.0 (2.0)	**0.002**
Hypertension, n (%)	210 (65.0)	139 (57.2)	71 (88.8)	**<0.001**
Hyperlipidemia, n (%)	283 (87.6)	211 (86.8)	72 (90.0)	0.45
Diabetes mellitus, n (%)	42 (13.0)	27 (11.1)	15 (18.8)	0.078
Current smoker, n (%)	74 (22.9)	49 (20.2)	25 (31.3)	**0.041**
Family history for CVD, n (%)	82 (25.4)	67 (27.6)	15 (18.8)	0.12
Established CVD, n (%)	114 (35.3)	64 (26.3)	50 (62.5)	**<0.001**
Coronary heart disease, n (%)	90 (27.9)	49 (20.2)	41 (51.2)	**<0.001**
Peripheral artery disease, n (%)	23 (7.1)	8 (3.3)	15 (18.8)	**<0.001**
Cerebrovascular disease, n (%)	26 (8.0)	16. (6.6)	10 (12.5)	0.092
hs-CRP, mg/dL	0.17 (0.3)	0.16 (0.3)	0.19 (0.3)	0.448
NGAL, µg/L	67.0 (30.1)	63.0 (28.2)	81.9 (33.2)	**<0.001**
Total cholesterol, mg/dL	195.0 (61.8)	201.0 (61.5)	175.0 (45.0)	**<0.001**
LDL cholesterol, mg/dL	116.0 (53.0)	122.0 (55.0)	104.0 (42.0)	**<0.001**
HDL cholesterol, mg/dL	58.0 (25.0)	61.0 (26.0)	52.0 (21.0)	**0.001**
Triglycerides, mg/dL	129.0 (88.8)	126.0 (80.5)	138.0 (107.0)	0.08
eGFR, mL/min/1.73m^2^	76.2 (±15.8)	76.6 (21.6)	73.8 (24.7)	0.061
BMI, kg/m^2^	25.4 (4.8)	25.3 (5.0)	25.6 (4.2)	0.46
Systolic BP, mmHg	120.5 (26.5)	117.5 (24.5)	126.3 (34.3)	**0.003**
Antihypertensive medication, n (%)	186 (57.6)	137 (56.4)	49 (61.3)	0.44
Statins or other lipid lowering medication, n (%)	178 (55.1)	132 (54.3)	46 (57.5)	0.62
High-intensity Statins, (n%)	62 (19.2)	41 (16.9)	21 (26.3)	0.07
Moderate-intensity Statins, (n%)	101 (31.3)	67 (27.6)	34 (42.5)	**0.012**
Low-intensity Statins, (n%)	6 (1.9)	5 (2.1)	1 (1.3)	0.64
Antiplatelet medication, n (%)	142 (44.0)	101 (41.6)	41 (51.2)	0.13
Antidiabetic medication, n (%)	35 (10.8)	22 (9.1)	13 (16.3)	0.07
Insulin, n (%)	8 (2.5)	7 (2.9)	1 (1.3)	0.42
Metformin therapy, n (%)	27 (8.4)	16 (6.6)	11 (13.8)	**0.045**

BP—blood pressure; BMI—body mass index; CVD—cardiovascular disease; HDL—high density lipoprotein; LDL—low density lipoprotein; GFR—glomerular filtration rate. For continuous variables, median (interquartile range) or mean values ± standard deviation are shown. For categorical variables, numbers (percentages) are displayed. *p*-values < 0.05 were considered statistically significant and are shown in bold.

**Table 2:** Measured parameters.

Measured Parameters *	Study Population (*n* = 323)	Low Total PV (*n* = 243)	High Total PV (*n* = 80)	*p-*Value
Total PV, mm^3^	250.0 (426.0)	163.0 (263.0)	732.5 (363.0)	**<0.001**
Carotid PV, mm^3^	76.0 (211.0)	39.0 (115.0)	351.0 (356.8)	**<0.001**
Femoral PV, mm^3^	144.0 (253.0)	75.0 (189.0)	463.0 (326.8)	**<0.001**
Carotid IMT, mm	0.72 (0.19)	0.71 (0.18)	0.79 (0.15)	**<0.001**
Ankle-brachial index	0.91 (0.14)	0.92 (0.13)	0.89 (0.14)	**0.004**
Pulse wave velocity, m/s	6.27 (2.42)	5.90 (2.28)	6.52 (2.67)	**0.029**

IMT—intima media thickness; PV—plaque volume. * Parameters are median (interquartile range) or mean ± standard deviation as indicated. *p*-values < 0.05 were considered statistically significant and are displayed in bold.

**Table 3:** Multivariate Regression for total PV.

Parameter	*b*	*p*
Total Plaque Volume (cPV)		
Age	0.40	<0.001
NGAL	0.28	<0.001
Established CVD	0.24	<0.001
Male Gender	0.21	<0.001
Smoking	0.18	<0.001
GFR	0.11	0.042

Model quality: cPV: Adjusted R^2^ = 0.38; *p* < 0.001; Relative contributions are given by the standardized regression coefficient *b.* Only variables with *p* < 0.05 are shown. PV—plaque volume; CVD—cardiovascular disease; GFR—glomerular filtration rate; NGAL—neutrophil gelatinase-associated lipocalin.

## Supplementary Materials

The following are available online at https://www.mdpi.com/2077-0383/9/9/2811/s1, Figure S1: (a,b) The higher the plaque load, the more frequently moderately potent to highly potent statins were prescribed. Our analyses showed that there was no statistical difference regarding NGAL levels between patients receiving statins and those who were statin naive. However, we know that statins can negatively affect NGAL levels and therefore the NGAL levels can be falsely low and could be higher if those patients were not on statins. Figure S2: NGAL levels were comparable between patients receiving metformin and those who were metformin naïve. The percentage of patients taking metformin was balanced between the high and low PV group. Figure S3: We also tested for IL-6 and IL-1beta in our study population. Interestingly, there was no statistical significance between the low and high PV group regarding plasma levels.

## Abbreviations

ABI	ankle brachial index
AUC	area under the curve
BMI	body mass index
BP	blood pressure
CVD	cardiovascular disease
CVRF	cardiovascular risk factor
CVRFs	cardiovascular risk factors
eGFR	estimated glomerular filtration rate
HDL	high density lipoprotein
hs-CRP	high sensitivity C reactive protein
IMT	intima media thickness
LDL	low density lipoprotein
NGAL	neutrophil gelatinase-associated lipocalin
PV	plaque volume
SD	standard deviation
