# Supplementary material for: NGAL Correlates with Femoral and Carotid Plaque Volume Assessed by Sonographic 3D Plaque Volumetry
Source: J Clin Med. 2020 Aug 31;9(9):2811. doi: 10.3390/jcm9092811 (PMC7565934; doi:10.3390/jcm9092811)
Supplement: Supplementary file 1 [file jcm-09-02811-s001.pdf]

Journal of
Clinical Medicine

Supplementary Materials

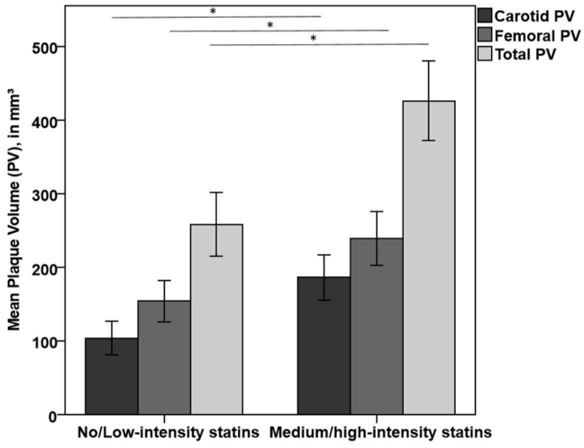

(a)

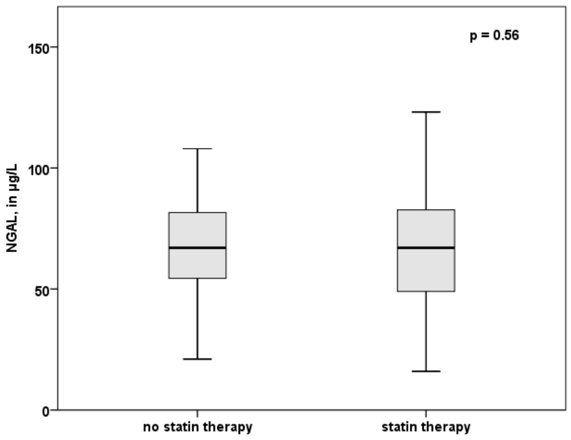

(b)

Figure S1. (**a,b**)The higher the plaque load, the more frequently moderately potent to highly potent statins were prescribed. Our analyses showed that there was no statistically difference regarding NGAL levels between patients receiving statins and those wo were statin naive. However, we know that statins can negatively affect NGAL levels and therefore the NGAL levels can be falsely low and could be higher if those patients were not on statins.

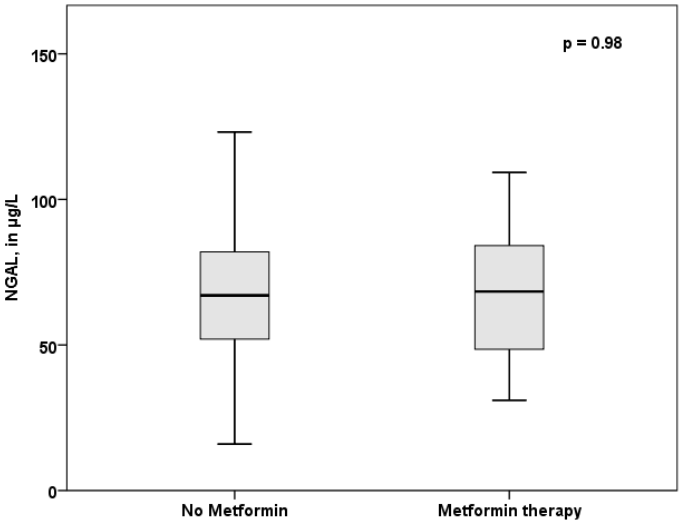

Figure S2. NGAL levels were comparable between patients receiving metformin and those who were metformin naïve. The percentage of patients taking metformin was balanced between the high and low PV group.

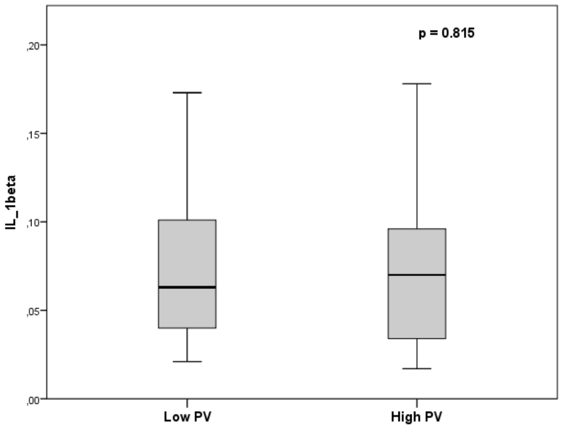

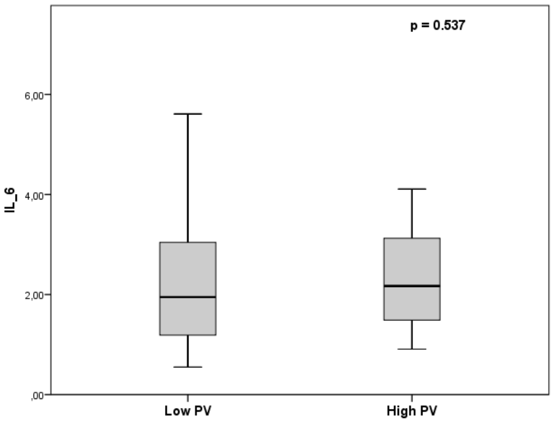

Figure S3. We also tested for IL-6 and IL-1beta in our study population. Interestingly, there was no statistically significance between the low and high PV group regarding plasma levels.
